# The challenge of a late diagnosis of Autism Spectrum Disorder: co-occurring trajectories and camouflage tendencies. a case report of a young Autistic female with Avoidant Restrictive Food Intake Disorder

**DOI:** 10.3389/fpsyt.2024.1447562

**Published:** 2025-01-24

**Authors:** Sara Passarini, Silvia Guerrera, Maria Picilli, Elisa Fucà, Laura Casula, Deny Menghini, Sabine Pirchio, Valeria Zanna, Giovanni Valeri, Stefano Vicari

**Affiliations:** ^1^ Psychology Unit, Child and Adolescent Neuropsychiatry Unit, Bambino Gesù Children’s Hospital, IRCCS, Rome, Italy; ^2^ Department of Dynamic and Clinical Psychology and Health Studies, Sapienza University of Rome, Rome, Italy; ^3^ Child and Adolescent Neuropsychiatry Unit, Bambino Gesù Children’s Hospital, IRCCS, Rome, Italy; ^4^ Child Neurology and Psychiatry Unit, Fondazione Policlinico Universitario Agostino Gemelli, IRCCS, Rome, Italy; ^5^ Department of Neuroscience, Catholic University of Sacred Heart, Rome, Italy; ^6^ Life Sciences and Public Health Department, Catholic University of Sacred Heart, Rome, Italy

**Keywords:** autism spectrum disorder, feeding and eating disorders, food selectivity, depressive mood, sensory profile

## Abstract

**Introduction:**

Autistic individuals may show several psychiatric co-occurrences, including Feeding and Eating Disorders (FEDs). Avoidant and Restrictive Food Intake Disorder (ARFID) consists of avoidance or restriction in food intake, leading to significant weight loss, nutritional deficiencies, and marked interference with psychosocial functioning. Both Autism Spectrum Disorder (ASD) and ARFID are characterized by the two main features of cognitive rigidity and sensory sensitivity, which may complicate differential diagnosis. There is a notable lack of information on the manifestation of ASD-ARFID co-occurrence, as well as tailored assessment tools and practice, and therapeutic approaches.

**Case description:**

This report provides a detailed description of L., a young girl with a late diagnosis of ASD who also developed unspecific depressive mood disorder and ARFID in co-occurrence. After the diagnosis of ASD, L. underwent multiple evaluations to address emerging psychiatric co-occurrences and symptom exacerbation, and in order to develop the most effective integrated treatment.

**Conclusion:**

The case of L. expands the knowledge on the phenotype of Autistic females and exemplifies how delayed diagnosis may exacerbate functioning differences and increase the camouflage phenomenon. Additionally, it underscores the importance of improving tailored evaluation, combined treatment plans, with both cognitive-behavioral therapy and drugs, and monitoring the evolving patterns of Autistic manifestations and associated psychiatric co-occurrences.

## 1 Introduction

Autism Spectrum Disorder (ASD) is a neurodevelopmental condition characterized by the early onset of neurodivergent characteristics in social communication along with restricted interests and repetitive behaviors (RRBs) (1, 2); its current global prevalence is estimated at around 1% (3). The diagnosis of ASD is often associated with a high likelihood of developing psychiatric co-occurrences such as anxiety disorders (estimated 30–39%), depressive disorder (estimated 15–21%), schizophrenia spectrum and other psychotic disorders (estimated 7–13%), suicidal ideation and attempts (estimated 1-66%), obsessive-compulsive disorder (estimated 7–10%), disruptive, impulse-control and conduct disorders (estimated 13–22%) and Attention Deficit Hyperactivity Disorder (estimated 28–46%) (4, 5). The prevalence rates of psychiatric co-occurrences in ASD may vary due to sample features (e.g. age), assessment methods, and individual cognitive abilities. Co-occurrences seem to persist from childhood to adolescence and adulthood (6, 7), worsening long-term outcomes, including increased mortality risk (6).

The age of Autistic individuals is a crucial for understanding developmental trajectories, with an intensification of psychiatric co-occurrences along the growth (8). Although co-occurring symptoms usually appear early in development, the co-occurring diagnosis is often missed until adolescence (9), due to diagnostic overshadowing (10). For instance, less engagement in social participation is often mistakenly attributed to social anxiety disorder, while specific interests are sometimes confused with obsessive-compulsive disorder (11). However, few studies have explored the characteristics and progression of co-occurring conditions in Autistic individuals (7, 12–14). Additionally, sex may affect co-occurring conditions, with females experiencing higher rates of depression, anxiety, suicidality, and eating disorders (15). For example, depressive symptoms tend to increase during adolescence in Autistic females than in males (7, 16).

Another common psychiatric co-occurrence associated with ASD is feeding and eating disorders (FED), with an estimated percentage of 20-46% (4). Currently, FEDs are conceptualized as a heterogeneous group of conditions such as anorexia nervosa, bulimia nervosa, binge eating disorder and avoidant restrictive food intake disorder (ARFID) (2). While anorexia nervosa or bulimia are characterized by obsessions with food, body weight, and shape, along with related thoughts and emotions that cause significant disturbances in a person’s eating behaviors (17), ARFID involves significant disruptions in nutritional and caloric intake that exceed typical variations in hunger, food preferences, or interest in eating (18). Hence, ARFID is more closely associated with disorders like pica and rumination (19–21). Further, differently from anorexia nervosa and bulimia nervosa, ARFID can only be diagnosed in the absence of concerns about body weight or shape. While individuals with anorexia nervosa fear caloric intake, ARFID concerns are often focused on the fear of choking during eating (22, 23). However, both anorexia nervosa and ARFID can present with strict food rules (24, 25), as well as low weight and malnutrition, necessitating a multidisciplinary approach (e.g. physicians, mental health professionals, dietitians, and family members) to restore weight (21).

Autistic individuals are more likely to experience eating issues compared to the general population (12, 13). Indeed, a substantial portion of Autistic children, approximately from 44% to 89%, experience feeding challenges (26). Meanwhile, people with FEDs seem to exhibit more Autistic traits (27, 28), with an estimated percentage of 8-37% for anorexia nervosa, and about 12.5% for ARFID (28).

ASD and FED co-occurrence may be explained by *selective eating*, described as a strong preference for specific food (e.g. sweets and calorie-dense food), typically grounded on foods’ color, shape, texture, or temperature (29). Further, Autistic children tend to show sensory differences and rigid patterns of mealtime behaviors, entailing feeding problems with fussy eating, limited food repertoire and amount of food consumed (5, 30). Other potential links between ASD and FEDs may be shared cognitive difficulties concerning underdeveloped theory of mind, emotional difficulties (e.g. empathy and alexithymia), set-shifting and cognitive inflexibility (28). Specifically, ARFID is more commonly co-occurring in Autistic children and adolescents compared to anorexia nervosa or bulimia, due to food selectivity and sensory sensitivity, which are main features of ARFID (31).

Focusing on ARFID, the condition involves avoiding foods for specific sensory-related aspects, fear of adverse consequences from feeding, and an overall lack of interest in eating, without any links to body image disturbances and fears of gaining weight (2). ARFID commonly generates adverse physical consequences and hampers the psychosocial functioning of individuals (2, 32, 33). ASD and ARFID may coexist, with an estimated percentage of 20% (34). Of note, ASD and ARFID co-occurrence may be explained by a symptomatology overlap characterized by cognitive rigidity and sensory sensitivity (33). Atypical sensory processing appears to characterize both ASD and ARFID, potentially moderating their interrelation. As a result, sensory inputs would be misinterpreted generating maladaptive behaviors (35) such as children’s overreaction to foods and eating aversion (36). Moreover, the presence of a diagnosis of ASD would enhance possibilities of meeting ARFID criteria, worsening eating difficulties rather than anticipating its onset (30). Studies on ARFID characterization in Autistic children pointed the sensory sensitivity as the most prevalent symptom (30). However, more searches are needed to look into ASD-ARFID co-occurrence focusing their presentation as well as assessment procedures and tools to grab their co-occurrence (30).

Based on the available evidence, we describe the case of a Caucasian female adolescent with a late diagnosis of ASD along with mood and feeding co-occurrences, who has been attending our outpatient units since September 2021.

## 2 Case description

### 2.1 Family history

L. has a twin brother, with unspecified learning disorders, and a younger brother. There is no known family history of psychiatric disorders; however, L’s parents reported a suspicion of obsessive-compulsive disorder in her maternal grandmother, who did not undergo formal evaluation.

### 2.2 Previous patient developmental and clinical history

L. is a Caucasian 14-year-old girl undergoing evaluation at the Child and Adolescent Psychiatry Unit of the Bambino Gesù Children’s Hospital, IRCCS due to progressively restricting her food intake, which is co-occurring with a diagnosis of ASD.

L. was born to non-related parents through a homologous *in vitro* fertilization twin pregnancy without complications. The amniocentesis had revealed a normal fetal karyotype. Delivery occurred via planned caesarean section at 36 weeks gestation (birth weight: 2240 grams; Apgar: 8-9). Her perinatal history was regular. Concerning neurodevelopmental history, independent walking emerged at 12 months and first words at 14 months. Later, L. showed a language disorder, specifically in the expressive domain, requiring speech therapy from 4.5 to 8 years. At 4 years, an audiometric examination was conducted displaying no irregularities. Since early childhood, she exhibited social interaction difficulties with peers, restricted interests, and selective eating with restricted range of foods.

In February 2020, at 10 years of age, L. underwent a cognitive diagnostic assessment in a private center with the Wechsler Intelligence Scale for Children- fourth edition (37), revealing an average cognitive functioning. A deeper investigation into Autistic traits was suggested as her family reported difficulties in peer relationships, hypersensitivity to noise, and selective eating. Mood fluctuations have been reported for about a year.

In August 2020, at 10 years, the investigation on Autistic traits was performed in a private center. L. underwent the Autism Diagnostic Observation Schedule-2 (38) revealing a calibrated severity score of 7/10. Clinicians highlighted the presence of restricted interests in stuffed toys, tendency towards isolation, frustration and irritability. The diagnosis of ASD in co-occurrence with an unspecified depressive disorder was made. Therapeutic prescriptions indicated weekly sessions of individual cognitive-behavioral therapy (CBT) combined with a psycho-educational group aimed at promoting communication strategies to support L. express their experiences, interests, feelings, and thoughts. Since September 2021, she exhibited a worsening of her clinical conditions with inappetence and severe weight loss of approximately 11 kg over nine months (from 58 kg to 47 kg). In June 2022, at 12 years, L. attended the Emergency Unit of Bambino Gesù Children’s Hospital, IRCSS, due to her serious weight loss, with a BMI of 18.8. L. denied purposely restrictive and dietary conduct and compensating behaviors. Her family reported persistent difficulties in relating with peers and mood fluctuations. They also reported frequent sleep irregularities characterized by difficulties in falling asleep. During the examination, clinicians pointed out cognitive rigidity, limited engagement in conversation, laconic speech, monotone tone, and Autistic traits. There were no misperceptions and suicidal ideation. The prescription for a deeper feeding evaluation was suggested.

### 2.3 Clinical observation findings

Sequentially, L. was referred to the Feeding and Eating Disorders Outpatient Unit of the Child and Adolescent Psychiatry Unit, Bambino Gesù Children’s Hospital, IRCSS for an overall neuropsychiatric assessment. **Table 1** provides the detailed assessment protocol. Based on DSM-5 criteria (54), a clinical diagnosis of ARFID was made in co-occurrence with ASD and unspecified depressive disorder. A nutritional plan was designed; prescriptions suggested individual CBT, family psychotherapy and parent training.

**Table 1:** Table of neuropsychological and psychopathological evaluations.

Neuropsychological and Psychopathological tools	First evaluation(June 2022)		Second evaluation (September 2022)	
**WISC-IV** (37)				
Total IQ index	Not performed		115	Average score
Verbal index			108	Average score
Performance index			126	High Average score
Working memory Index			103	Average score
**ADOS-2** (38)				
Social affect domain	Not performed		CSS= 7/10	
Restrictive and repetitive behaviors domain			CSS= 4/ 10	
Overall ADOS-2			CSS= 6/10	*Autism*
**ADI-R** (39)				
Reciprocal social interaction	Not performed		14/10	Cut off= 10
Communication			10/8	Cut off= 8
Restrictive and repetitive behaviors			1/3	Cut off= 3
Development			2/1	Cut off= 1
**SRS** (40)				
Total score	Not performed		T-score: >90	*Severe Profile*
Social awareness			T-score: 60	Mild score
Social cognition			T-score: 74	Moderate score
Social communication			T-score: >90	Severe score
Social motivation			T-score: >90	Severe score
Restrictive and repetitive behaviors			T-score: 88	Severe score
**ABAS-II** (41)				
Composite index	Not performed		Percentile: <1^st^	Extremely Low score
Conceptual domain			Percentile: <1^st^	Extremely Low score
Social domain			Percentile: <1^st^	Extremely Low score
Practical domain			Percentile: 3^rd^	Extremely Low score
**CBCL/ 6-18** (42, 43)				
**Competence scale scores**				
Activities	T-score: 47	Average score	T-score: 46	Average score
Social	T-score: 32	Average score	T-score: 25	Clinical score
School	Not computable		Not computable	
Total competence score	Not computable		Not computable	
**Syndrome scale scores**				
Anxious/ Depressed	T-score: 62	Average score	T-score: 74	Clinical score
Withdrawn/ Depressed	T-score: 93	Clinical score	T-score: 100	Clinical score
Somatic Complaints	T-score: 78	Average score	T-score:70	Clinical score
Social problems	T-score: 61	Average score	T-score: 68	Borderline score
Thought problems	T-score: 69	Average score	T-score: 75	Clinical score
Attention problems	T-score: 63	Average score	T-score: 68	Borderline score
Rule-breaking behavior	T-score: 57	Average score	T-score: 64	Average score
Aggressive behavior	T-score: 52	Average score	T-score: 67	Borderline score
**Internalizing, Externalizing, Total Problems, and Other Problems**				
Internalizing problems	T-score: 76	Clinical score	T-score: 78	Clinical score
Externalizing problems	T-score: 54	Average score	T-score: 67	Clinical score
Total problems	T-score: 67	Average score	T-score: 73	Clinical score
**DSM-Oriented Scales**				
Depressive problems	T-score: 77	Clinical score	T-score: 88	Clinical score
Anxiety problems	T-score: 55	Average score	T-score: 64	Average score
Somatic problems	T-score: 77	Clinical score	T-score: 66	Borderline score
Attention deficit	T-score: 52	Average score	T-score: 60	Average score
Oppositional defiant problems	T-score: 51	Average score	T-score: 67	Borderline score
Conduct problems	T-score: 51	Average score	T-score: 61	Average score
**2007 Scale scores**				
Sluggish Cognitive Tempo	T-score: 73 Clinical Score	Average score	T-score: 75	Clinical score
Obsessive Compulsive Problems	T-score: 66	Average score	T-score: 69	Borderline score
Stress Problems	T-score: 67	Average score	T-score: 82	Clinical score
**PSI** (44)				
Total parental distress	Percentile: 80^th^	Clinical score	Percentile: 80^th^	Clinical score
Parental Distress	Percentile: 90^th^	Clinical score	Percentile: 95^th^	Clinical score
Parent–Child Dysfunctional Interaction	Percentile: 95^th^	Clinical score	Percentile: 100^th^	Clinical score
Difficult Child	Percentile: 95^th^	Clinical score	Percentile: 100^th^	Clinical score
**ABC** (45)	Not performed		No clinical scores	
**MASC-2 (self-report)** (46)				
Anxiety probability:	1	*Borderline anxiety probability*	Not performed	
Total score	T-score: 50	Average score		
Separation anxiety/ phobias	T-score: 72	Clinical score		
Social anxiety	T-score: 44	Average score		
GAD index	T-score: 52	Average score		
Humiliation/Rejection	T-score: 40	Average score		
Performance fears	T-score: 53	Average score		
Obsessions and Compulsions	T-score: 43	Average score		
Physical symptoms	T-score: 59	Average score		
Panic	T-score: 66	Borderline score		
Tense/ Restless	T-score: 49	Average score		
Harm Avoidance	T-score: 40	Average score		
Inconsistency index	6	cut off>10		
**MASC-2 (parent-report)** (46)				
Anxiety probability:	Not performed		3	*Very Elevated anxiety probability*
Total score			T-score: 78	Clinical score
Separation anxiety/ phobias			T-score: 62	Borderline score
Social anxiety			T-score: 88	Clinical score
GAD index			T-score: 83	Clinical score
Humiliation/Rejection			T-score: 83	Clinical score
Performance fears			T-score: 84	Clinical score
Obsessions and Compulsions			T-score: 46	Average score
Physical symptoms			T-score: 81	Clinical score
Panic			T-score: 88	Clinical score
Tense/ Restless			T-score: 70	Clinical score
Harm Avoidance			T-score: 56	Average score
Inconsistency index			6	cut-off >8
**CDI-2 (parent-report)** (47)				
Total score	Not performed		T-score: 66	*Elevated overall score: more concerns than are typically reported*
Emotional problems			T-score: 74	Clinical score
Functional problems			T-score: 57	Average score
**CDI-2 (self-report)** (47)				
Total score	T-score: 68	*Elevated overall score: more concerns than are typically reported*	Not performed	
Emotional problems	T-score: 61	High average score		
Negative mood/ Physical symptoms	T-score: 64	High average score		
Negative self-esteem	T-score: 55	Average score		
Functional problems	T-score: 72	Clinical score		
Interpersonal problems	T-score: 57	High average score		
Ineffectiveness	T-score: 70	Clinical score		
**SDSC** (48)				
Disorders in initiating and maintaining sleep	Not performed		T-score: 60	Average score
Sleep breathing disorders			T-score: 45	Average score
Disorders of arousal nightmares			T-score: 47	Average score
Sleep wake transition disorders			T-score: 46	Average score
Disorders of excessive somnolence			T-score: 77	Clinical score
Sleep hyperhidrosis			T-score: 45	Average score
Total SDSC score			T-score: 58	Average score
**SSP-2** (49)	Not performed		The sensory processing pattern categorized into “*like others*”	
**YSR** (43)	Non scorable, incomplete compilation		Not performed	
**BUT** (50)	Non scorable, incomplete compilation		Not performed	
**EDI- 3** (51)	Non scorable, incomplete compilation		Not performed	
**K-SADS-PL (self and parent report)** (52)	-Diminished appetite and reduced interest in food, with avoidance of certain foods and occasional meal skipping. Neither body dysmorphia nor fear of gaining weight (from 11 years old). -Anhedonia, apathy, emotional instability, irritability, fatigue, difficulty concentrating, a lack of response to positive stimuli, and a propensity for self-evaluation and pessimistic thinking (from 12 years old).		- Persistent depressive mood with feelings of insecurity, low self-esteem, anxiety linked to social ineffectiveness, avoidance of social situations (from 10 years old). - Restrictive eating pattern (from 11 years old). - Intrusive thoughts concerning the fear of not being noticed by peers, daily mood swings, anhedonia, apathy, fatigue, and difficulty concentrating. Irritability and impulsivity, leading to self-cutting without specific triggers or suicidal ideation (from 12 years old).	
**CGAS** (53)	Clinician rating:55/100	*Moderate functioning impairments in more than one area*	Clinician rating:55/100	*Moderate functioning impairments in more than one area*

WISC- IV, Wechsler Intelligence Scale for Children-Fourth Edition (37); ADOS-2, Autism Diagnostic Observation Schedule 2 (38); CSS, calibrated severity score; ADI-R, Autism Diagnostic Interview-Revised (39); SRS-2, Social Responsiveness Scale, second edition (40); ABAS II, The Adaptive Behavior Assessment System Second Edition version (41); CBCL/ 6-18, Child Behavior Checklist7 6-18 (42, 43); PSI, Parenting Stress Index (44); ABC, Aberrant behavior checklist (45); MASC-2, Multidimensional Anxiety Scale for Children-Second Edition (46); CDI-2, Children's Depression Inventory 2 (47); SDSC, Sleep Disturbances Scale for Children (48); SSP-2, Short Sensory Profile -2 (49); YSR, Youth Self-Report (43); BUT, Body Uneasiness Test (50); EDI-3, Eating Disorder Inventory III (51); K-SADS-PL, Kiddie Schedule for Affective Disorders and Schizophrenia for School-Aged Children Present-Lifetime version DSM-5 (52); CGAS, Children’s Global Assessment Scale (53).

In September 2022, L. attended the ASD Outpatient Unit of the Child and Adolescent Psychiatry Unit, Bambino Gesù Children’s Hospital, IRCSS for further evaluations (**Table 1**). Based on clinical observation and neuropsychological assessment, the diagnosis of ASD was confirmed in co-occurrence with ARFID and unspecified depressive disorder without any genetic condition associated. Arrangements for both individual CBT and group psychotherapy sessions were reaffirmed to provide a comprehensive and supportive therapeutic plan.

#### 2.3.1 Neuropsychological and psychopathological assessment

In June 2022, a psychopathological assessment including the Kiddie Schedule for Affective Disorders and Schizophrenia (52) was conducted, alongside parent-report questionnaires, including the Child Behavior Checklist (42), the Children’s Depression Inventory 2 (47), and the Multidimensional Anxiety Scale for Children-Second Edition (46) to detect potential behavioral and emotional co-occurrences. Finally, the Parenting Stress Index (44) was also proposed to her parents.

In September 2022, the previous evaluation was expanded to include an investigation of cognitive abilities, using the Wechsler Intelligence Scale for Children-Fourth Edition (37), and Autistic traits through the Autism Diagnostic Observation Schedule-2/module 3 (38), as well as the Autism Diagnostic Interview-Revised (39).

**Table 1** provides a detailed overview of neuropsychological and psychopathological evaluations conducted in June and September 2022.

#### 2.3.2 Genetic analysis

In September 2022, L. underwent genetic tests such as Chromosomal Microarray Analysis, FMR1 gene analysis, and karyotype, which did not display any variations.

### 2.4 Therapeutic intervention, follow-up, and outcomes

Due to the worsening of her symptomatology with persistent irritability, easy impulsivity, restlessness, emotional dysregulation and notable social withdrawal, the need for drug therapy with a new generation antipsychotic was discussed with the family. In October 2022, aripiprazole (2.5 mg/day) was administered and combined with the previous psychological interventions.

At the follow-up in December 2022, no improvements were noted and a booster therapy was chosen, increasing the aripiprazole dosage to 3.75 mg/day.

At the follow-up in March 2023, L. showed a persistent lack of peer interaction at school, despite some participation in group activities. Her parents reported enhancements in communicative intentionality, mainly with adults. Although daily nutrition has been regulated, selective food intake based on limited taste preferences persisted. Parents reported that individual CBT began in January 2023, accompanied by participation in group therapy but with limited engagement. Despite good compliance with drug therapy, L. expressed concerns about experiencing drowsiness as a side effect. Of note, L. exhibited a greater inclination toward dialogue with clinicians, displaying an increased willingness to answer questions and engage in future planning. L. expressed experiencing anxiety during school performances and the fear of being judged by others. Additionally, she kept on encountering ongoing difficulties falling asleep. Through clinical interviews, we substantiated the presence of a depressive mood with impulsive behavior. Hence, the daily dosage of aripiprazole was increased to 5 mg/day.

At the follow-up in January 2024, L. appeared clear-minded, oriented, and well groomed. L. showed less social withdrawal with greater openness to dialogue, actively engaging with clinicians. However, challenges remained in understanding her own emotions and in finding closure on certain topics directly affecting her. Her mood was stable. Pharmacological and psychotherapy plans were reaffirmed without changes.

**Figure 1:**
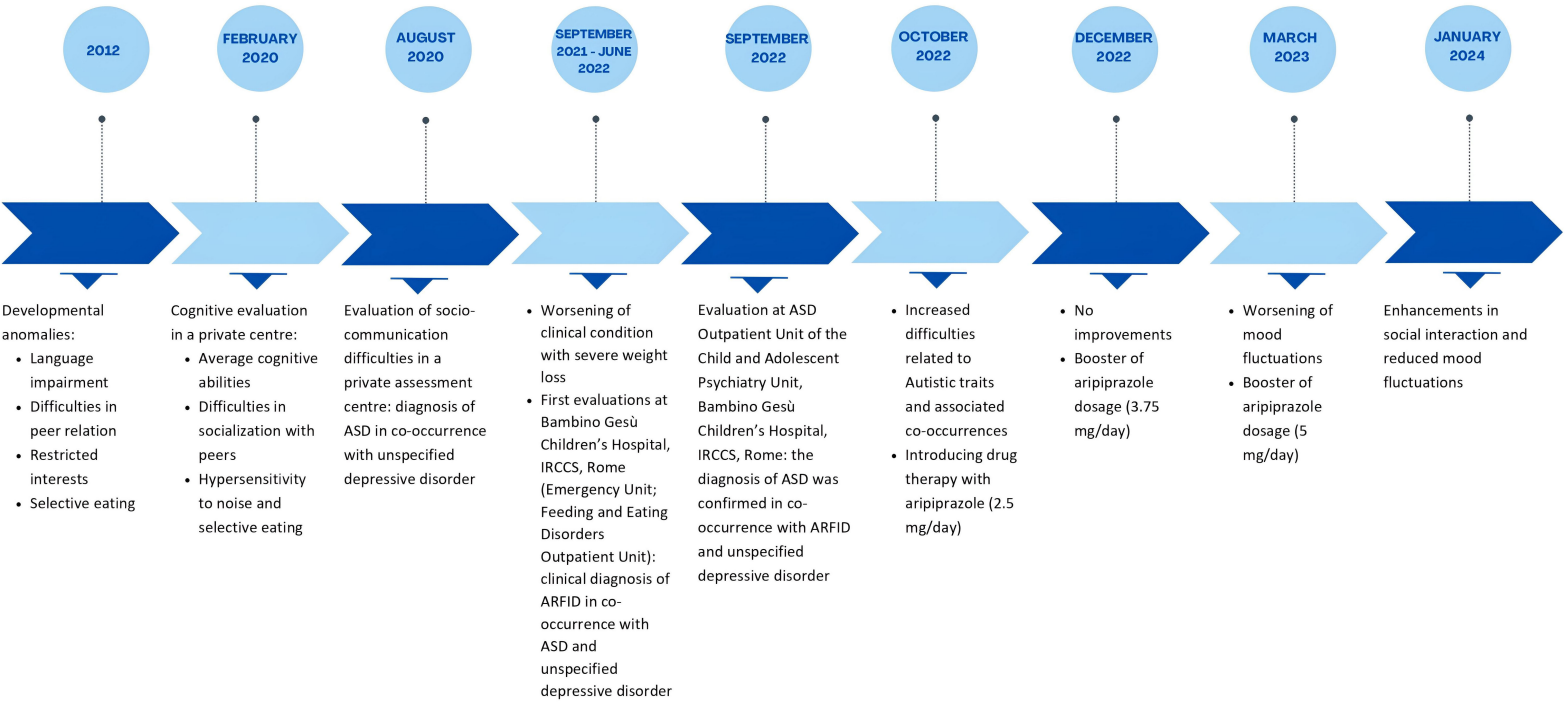
Timeline summarizing the symptomatology and assessment history of L.

**Figure 1** illustrates the timeline of L.’s evaluation history.

## 3 Discussion

The aim of this paper is to point the evolutionary trajectory of Autistic characteristics in the case of a late diagnosis, showing how delays in the diagnostic process may exacerbate Autistic traits and foster the onset of psychiatric co-occurrences over time (55–57). The advancement of well-being is a fundamental right for all individuals, even in the presence of a pathological condition such as neurodevelopmental disorders (NDDs). To enhance the adaptive functioning and quality of life for individuals with NDDs, a multidisciplinary approach to care is mandatory, addressing both the primary pathology and any co-occurrences and aiming to foster timing diagnosis as they are associated with better outcomes, timely interventions, and reduced burden of the condition and co-occurrences (58, 59).

However, timing evaluations are not always performed. Indeed, underdiagnosed states often rise to clinicians’ attention during adolescence, when social demands on individuals gradually increase, exacerbating underlying discomfort by either amplifying an already present but less apparent symptomatology. Possibly the adolescence stage would also uphold the development of additional co-occurring symptoms, which may or not be specific to further and peculiar psychopathological conditions.

There is a linear relation between the age of the diagnosis of ASD and mental health co-occurrences, with children diagnosed around 11 years showing the strongest association with other mental health issues (60). This may result from delayed evidence-based treatments and insufficient comprehensive care, potentially hindering the development of adequate coping skills and social support (60, 61). Additionally, the late diagnosis of ASD is more common for females without a learning disability (11). This scenario is linked with the “camouflage hypothesis” (62) for which the attitude of masking Autistic traits, within the presence of internalizing problems, such as depressive and eating disorders, are more common in the phenotype of Autistic females than in males (63, 64). However, research on Autistic females is still scarce (65), generating challenges for a good clinical practice assessment and intervention.

The described case exemplifies this background.

Firstly, L. received a late diagnosis of ASD at age 10, potentially delaying appropriate therapeutic interventions and the identification of co-occurring conditions. Mood and feeding co-occurrences may have emerged due to the late diagnosis of ASD; the lack of support in presence of Autistic characteristics could have led to elevated stress levels and worsened overall functioning, negatively affecting her well-being. As a result, L. has done a multi-psychopathological pathway. Of note, given her good cognitive abilities it’s possible that L.’s could have tried to mask her Autistic characteristics using camouflage, a common phenomenon in Autistic females (66). If camouflaging could have helped L. in managing everyday challenges, the same tendency might have structured the Autistic traits into multiple co-occurrences with an overtime worsening. Even though strong cognitive skills may provide a greater set of resources for undergoing CBT therapy, potentially leading to more positive effects on psychopathology outcomes (67). Perhaps, in this case, good cognitive abilities may allow for a greater insight capacity with high awareness of one’s own difficulties, leading to a sense of inadequacy and continuous attempts at camouflaging. As seen, L. first developed a transient form of ARFID, with severe weight loss, and then a clear unspecified depressive disorder with Non-Suicidal Self-Injury probably linked to her difficulties in peer relation. It is crucial to pay attention to depressive states in co-occurrence with ASD. Females with strong cognitive skills not only tend to remain frequently undiagnosed but also to manifest increased mood co-occurrences and a higher risk of self-injury (11). The lack of coping strategies in individuals with a similar manifestation of the Spectrum condition may exacerbate social withdrawal. Hence, we decided to tailor an integrated treatment plan for L., combining CBT, psychoeducation and pharmacological intervention.

Specifically, therapeutic prescriptions indicated CBT for facing mood (68–73) and feeding (12, 13, 72, 73) co-occurrences. To address L.’s mood difficulties, it was recommended to focus directly on her emotional processes in a proactive and solution-oriented manner. The goal was to improve her self-regulation, enhance her sense of personal efficacy, strengthen her problem-solving abilities, and cultivate a sense of enjoyment and mastery (69). In addition, to manage L.’s food selectivity, an intervention was recommended to help the Autistic girl develop personalized CBT strategies, including self-monitoring, gradual reintroduction of feared foods, and identifying triggers to overcome feeding difficulties (73, 74).

Aiming at embracing a person-centered approach, the therapeutic plan also included psychoeducational sessions which emphasized prioritizing and valuing L’s experiences, needs, preferences, passions, pursuits, and desires of the Autistic individual (14, 75, 76).

The pharmacological plan was based on the current literature evidence underlining risperidone and aripiprazole as elective drugs for ASD with psychiatric co-occurrences, behavioral problems and RRBs (77, 78). Indeed, aripiprazole, approved by the U.S. Food and Drug Administration for children and adolescents aged from 6 to 17 years to address irritability associated with Autistic traits, may provide an effective short-term pharmacological option for managing specific behavioral co-occurrences (79, 80). Although the best practice intervention for NDDs, such as ASD, would be CBT (81), evaluating the introduction of a pharmacological plan is also crucial. An integrated approach combining pharmacological and psychotherapeutic interventions may be more effective than using either intervention alone, particularly in cases with co-occurrences (82, 83). For instance, the treatment plan of Autistic characteristics with mood co-occurrences should combine non-medical interventions, like CBT, and pharmacological treatments. Similarly, in the case of ARFID co-occurrence with ASD, integrating drugs such as antidepressants, antipsychotics, and stimulants with CBT is recommended. However, evidence remains limited, especially for Autistic adolescents and adults and other psychiatric co-occurrences (83).

Concerning ASD and ARFID co-occurrence, there is a gap in the literature exploring their interrelation, leading to a scarcity of solid data. Hence, epidemiological studies, a focus on the manifestation of co-occurrences, and treatment trials should be promoted. As both conditions tend to show an atypical sensory processing as a prevalent feeding feature, it may be particularly challenging for clinicians carrying a differential diagnosis. Available tools are not tailored for it, generating difficulties during the assessment. The Childhood Autism Rating Scale, 2nd Edition (84) and the Short Sensory Profile-2 (49) may allow for a differential investigation of sensory aspects providing an initial support to healthcare workers. For instance, in the aforementioned case, the Short Sensory Profile-2 (49) supported the clinical diagnostic process confirming a clear and distinct FED not attributable to ASD sensitivity. The measure is tailored to catch the peculiar sensory processing pattern of individuals, explaining how information are processed and their behavioral correlates (49). As L.’s sensory profile encompasses the range of “like most people”, it confirms that her food selectivity was not related to the Autistic characteristics but to a clear feeding issue. Hence, the diagnosis of ARFID was timely appropriate to correctly manage her feeding condition. Given the potential overlaps between ASD and ARFID, the decision for a more in-depth investigation into the Spectrum condition was carefully made. This approach also allowed for targeted interventions aimed at preventing future psychiatric co-occurrences.

Of note, L.’s case could be read through the lens of the neurodevelopmental continuum paradigm, suggesting ASD and other psychiatric disorders may be understood as part of a pathological continuum pathway (85). This theory holds particular significance for Autistic individuals with low support needs and females with peculiar camouflaging tendencies (86). As it may have happened with L., the ongoing effort to mask the Autistic traits and related issues could result in the development of new co-occurrences connected to a profound distress inherent in camouflaging.

The case of L. underscores the importance of implementing research on the manifestation of ASD with a specific focus on adolescence, a developmental stage characterized by an intrinsic vulnerability regardless the presence of an NDD. Further, as the guidelines for ASD of the National Institute for Health and Care Excellence exhort (87), L.’s case highlights the importance of tailored and integrated therapeutic plans based on both pharmacological treatment and psychotherapy. Gold standard diagnostic process and treatment plan should be personalized and run with a holistic perspective that acknowledges the interconnection of psychiatric disorders. The above-mentioned case emphasizes the need for longitudinal research with adequately sized samples to identify evolving patterns of co-occurring symptomatology in a preventive perspective with the aims of implementing a lifespan support and preventing confirmed psychiatric co-occurrences.

The peculiar features of the described symptomatic evolution and the comprehensive assessment seem to be points of strength of the current report. However, the main limitations are the paucity of follow-up evaluations over time and of comprehensive information about L.’s early developmental history. Monitoring any potential shifts in L.’s Autistic traits and psychiatric co-occurrences is essential to sustain tailored interventions suited to age-related symptoms. Hence, a longitudinal approach is essential to comprehensively understand the symptomatology pattern of this specific case.

Finally, we would like to support and embrace the hypothesis of a delayed diagnosis of ASD as a “fertile ground” for psychiatric co-occurrences, highlighting the decisive importance of assuming a prospective view throughout conditions rather than categorizing them into clear distinguished categories. It is also fundamental to restate that any assessment and treatment plan should be tailored to the manifestation of Autistic traits in relation to age. More studies are required for better understanding the clinical features and the optimal treatment combination for the spectrum condition in presence of psychiatric co-occurrences, especially in Autistic females without learning difficulties.

## Data Availability

The original contributions presented in the study are included in the article/supplementary material. Further inquiries can be directed to the corresponding author.

## References

[1] Salari N Rasoulpoor S Rasoulpoor S Shohaimi S Jafarpour S Abdoli N. The global prevalence of autism spectrum disorder: a comprehensive systematic review and meta-analysis. Ital J Pediatr. (2022) 48:112. doi: 10.1186/s13052-022-01310-w 35804408 PMC9270782

[2] American Psychiatric Association. Diagnostic and statistical manual of mental disorders, 5th ed. (2022). doi: 10.1176/appi.books.9780890425787

[3] Zeidan J Fombonne E Scorah J Ibrahim A Durkin MS Saxena S. Global prevalence of autism: A systematic review update. Autism Res Off J Int Soc Autism Res. (2022) 15:778−90. doi: 10.1002/aur.v15.5 PMC9310578 35238171

[4] Micai M Fatta LM Gila L Caruso A Salvitti T Fulceri F. Prevalence of co-occurring conditions in children and adults with autism spectrum disorder: A systematic review and meta-analysis. Neurosci Biobehav Rev déc. (2023) 155:105436. doi: 10.1016/j.neubiorev.2023.105436 37913872

[5] Parsons MA. Autism diagnosis in females by eating disorder professionals. J Eat Disord 11 mai. (2023) 11:73. doi: 10.1186/s40337-023-00785-0 PMC10173598 37170136

[6] Lai MC Kassee C Besney R Bonato S Hull L Mandy W. Prevalence of co-occurring mental health diagnoses in the autism population: a systematic review and meta-analysis. Lancet Psychiatry. (2019) 6:819−29. doi: 10.1016/S2215-0366(19)30289-5 31447415

[7] McCauley JB Elias R Lord C. Trajectories of co-occurring psychopathology symptoms in autism from late childhood to adulthood. Dev Psychopathol. (2020) 32:1287−302. doi: 10.1017/S0954579420000826 32677592 PMC7655668

[8] Fucà E Guerrera S Valeri G Casula L Novello RL Menghini D. Psychiatric comorbidities in children and adolescents with high-functioning autism spectrum disorder: A study on prevalence, distribution and clinical features in an Italian sample. J Clin Med. (2023) 12:677. doi: 10.3390/jcm12020677 36675606 PMC9864301

[9] Aggarwal S Angus B. Misdiagnosis versus missed diagnosis: diagnosing autism spectrum disorder in adolescents. Australas Psychiatry Bull R Aust N Z Coll Psychiatr. (2015) 23:120−3. doi: 10.1177/1039856214568214 25653302

[10] Simonoff E Pickles A Charman T Chandler S Loucas T Baird G. Psychiatric disorders in children with autism spectrum disorders: prevalence, comorbidity, and associated factors in a population-derived sample. J Am Acad Child Adolesc Psychiatry. (2008) 47:921−9. doi: 10.1097/CHI.0b013e318179964f 18645422

[11] Gupta N Gupta M. Diagnostic overshadowing in high-functioning autism: mirtazapine, buspirone, and modified cognitive behavioral therapy (CBT) as treatment options. Cureus. mai. (2023) 15:e39446. doi: 10.7759/cureus.39446 PMC10289477 37362512

[12] Price T Apostolopoulou T Jones K. Virtually delivered cognitive behavioural therapy for avoidant restrictive food intake disorder (CBT-AR): a case study in an adult with elevated autistic traits. Eat Disord. (2024), 1–21. doi: 10.1080/10640266.2024.2346372 38695293

[13] Proctor KB Volkert VM Klin A Vickery BP Sharp WG. The intersection of autism spectrum disorder, food allergy, and avoidant/restrictive food intake disorder: A clinical case study. J Pediatr. (2024) 269:113965. doi: 10.1016/j.jpeds.2024.113965 38369235

[14] Sakamoto S Miyawaki D Goto A Harima Y Tokuhara D Inoue K. COVID-19 phobia in a boy with undiagnosed autism spectrum disorder: A case report. Med (Baltimore). (2021) 100:e26233. doi: 10.1097/MD.0000000000026233 PMC8183832 34087907

[15] Secci I Petigas L Cuenod A Klauser P Kapp C Novatti A. Case report: Treatment-resistant depression, multiple trauma exposure and suicidality in an adolescent female with previously undiagnosed Autism Spectrum Disorder. Front Psychiatry. (2023) 14:1151293. doi: 10.3389/fpsyt.2023.1151293 37181890 PMC10169628

[16] Gotham K Unruh K Lord C. Depression and its measurement in verbal adolescents and adults with autism spectrum disorder. Autism. (2015) 19:491−504. doi: 10.1177/1362361314536625 24916450 PMC4467786

[17] National Institute of Mental Health: Eating Disorders. (2016). Available online at: https://www.nimh.nih.gov/health/topics/eating-disorders.

[18] A systematic review and meta-analysis of intensive multidisciplinary intervention for pediatric feeding disorders: how standard is the standard of care? Available online at. 10.1016/j.jpeds.2016.10.002 27843007

[19] Nicely TA Lane-Loney S Masciulli E Hollenbeak CS Ornstein RM. Prevalence and characteristics of avoidant/restrictive food intake disorder in a cohort of young patients in day treatment for eating disorders. J Eat Disord. (2014) 2:21. doi: 10.1186/s40337-014-0021-3 25165558 PMC4145233

[20] Forman SF McKenzie N Hehn R Monge MC Kapphahn CJ Mammel KA. Predictors of outcome at 1 year in adolescents with DSM-5 restrictive eating disorders: report of the national eating disorders quality improvement collaborative. J Adolesc Health Off Publ Soc Adolesc Med. (2014) 55:750−6. doi: 10.1016/j.jadohealth.2014.06.014 25200345

[21] Kennedy GA Wick MR Keel PK. Eating disorders in children: is avoidant-restrictive food intake disorder a feeding disorder or an eating disorder and what are the implications for treatment? F1000Research. (2018) 7:88. doi: 10.12688/f1000research 29399331 PMC5773930

[22] Ornstein RM Essayli JH Nicely TA Masciulli E Lane-Loney S. Treatment of avoidant/restrictive food intake disorder in a cohort of young patients in a partial hospitalization program for eating disorders. Int J Eat Disord. (2017) 50:1067−74. doi: 10.1002/eat.22737 28644568

[23] Peebles R Lesser A Park CC Heckert K Timko CA Lantzouni E. Outcomes of an inpatient medical nutritional rehabilitation protocol in children and adolescents with eating disorders. J Eat Disord. (2017) 5:7. doi: 10.1186/s40337-017-0134-6 28265411 PMC5331684

[24] Lucarelli J Pappas D Welchons L Augustyn M. Autism spectrum disorder and avoidant/restrictive food intake disorder. J Dev Behav Pediatr JDBP. (2017) 38:79−80. doi: 10.1097/DBP.0000000000000362 27824638

[25] Katzman DK Stevens K Norris M. Redefining feeding and eating disorders: What is avoidant/restrictive food intake disorder? Paediatr Child Health. (2014) 19:445−6. doi: 10.1093/pch/19.8.445 25383003 PMC4220532

[26] Lee M Lee S Sohn JW Kim KW Choi HJ. Assessment methods for problematic eating behaviors in children and adolescents with autism spectrum disorder. Soa–Chongsonyon Chongsin Uihak J Child Adolesc Psychiatry. (2024) 35:57−65. doi: 10.5765/jkacap.230065 PMC10774564 38204745

[27] Carter Leno V Micali N Bryant-Waugh R Herle M. Associations between childhood autistic traits and adolescent eating disorder behaviours are partially mediated by fussy eating. Eur Eat Disord Rev J Eat Disord Assoc. (2022) 30:604−15. doi: 10.1002/erv.2902 PMC9542277 35388530

[28] Inoue T Otani R Iguchi T Ishii R Uchida S Okada A. Prevalence of autism spectrum disorder and autistic traits in children with anorexia nervosa and avoidant/restrictive food intake disorder. Biopsychosoc Med. (2021) 15:9. doi: 10.1186/s13030-021-00212-3 34001197 PMC8130445

[29] Peretti S Mariano M Mazzocchetti C Mazza M Pino MC Verrotti Di Pianella A. Diet: the keystone of autism spectrum disorder? Nutr Neurosci. (2019) 22:825−39. doi: 10.1080/1028415X.2018.1464819 29669486

[30] Bourne L Mandy W Bryant-Waugh R. Avoidant/restrictive food intake disorder and severe food selectivity in children and young people with autism: A scoping review. Dev Med Child Neurol. (2022) 64:691−700. doi: 10.1111/dmcn.15139 35112345

[31] Cermak SA Curtin C Bandini LG. Food selectivity and sensory sensitivity in children with autism spectrum disorders. J Am Diet Assoc. (2010) 110:238−46. doi: 10.1016/j.jada.2009.10.032 20102851 PMC3601920

[32] World Health Organization. Feeding and eating disorders. In: International Statistical Classification of Diseases and Related Health Problems. 11th edn. Geneva, Switzerland: WHO (2020).

[33] Farag F Sims A Strudwick K Carrasco J Waters A Ford V. Avoidant/restrictive food intake disorder and autism spectrum disorder: clinical implications for assessment and management. Dev Med Child Neurol. (2022) 64:176−82. doi: 10.1111/dmcn.14977 34405406

[34] Koomar T Thomas TR Pottschmidt NR Lutter M Michaelson JJ. Estimating the prevalence and genetic risk mechanisms of ARFID in a large autism cohort. Front Psychiatry. (2021) 12:668297. doi: 10.3389/fpsyt.2021.668297 34177659 PMC8221394

[35] Calisan Kinter R Ozbaran B Inal Kaleli I Kose S Bildik T Ghaziuddin M. The sensory profiles, eating behaviors, and quality of life of children with autism spectrum disorder and avoidant/restrictive food intake disorder. Psychiatr Q. (2024) 95:85−106. doi: 10.1007/s11126-023-10063-6 38085408

[36] Sari SA. Coexistence of autism and eating disorder: a case report. Dusunen Adam J Psychiatry Neurol. (2018) 31:301. doi: 10.5350/DAJPN2018310308

[37] Wechsler D. Wechsler intelligence scale for children – Fourth edition (WISC-IV). San Antonio, TX: The Psychological Corporation (2003).

[38] Lord C Rutter M DiLavore PC Risi S Gotham K Bishop S. Autism Diagnostic Observation Schedule (ADOS-2)–, 2nd Edn. Los Angeles: Western Psychological Services.

[39] Lord C Rutter M Le Couteur A. Autism Diagnostic Interview-Revised: a revised version of a diagnostic interview for caregivers of individuals with possible pervasive developmental disorders. J Autism Dev Disord. (1994) 24:659−85. doi: 10.1007/BF02172145 7814313

[40] Constantino JN Scale GSR. (SRS-2). Los Angeles, CA: Western Psychological Services (2012).

[41] Harrison PL Oakland T. ABAS II Adaptive Behavior Assessment System. 2nd ed. Florence: Giunti Psychometrics (2014).

[42] Achenbach TM Rescorla LA. Manual for the ASEBA school-age forms & Profiles. Burlington, VT: University of Vermont, Research Center for Children, Youth, & Families (2001).

[43] Achenbach TM Dumenci L Rescorla LA. Ratings of relations between DSM-IV diagnostic categories and items of the CBCL/6-18, TRF, and YSR. Burlington, VT: University of Vermont (2001) p. 1–9. p.

[44] Abidin RR. Parenting Stress Index: a measure of the parent–child system. In: Woods RJ Zalaquett CP, Eds., Evaluating Stress: A Book of Resources, Scarecrow Press, Lanham, 227–91.

[45] Aman MG Singh NN Stewart AW Field CJ. Psychometric characteristics of the aberrant behavior checklist. Am J Ment Defic. (1985) 89:492−502. 3158201

[46] March JS. Multidimensional anxiety scale for children 2nd edition (MASC 2). Toronto: Multi-Health Systems Inc (2013).

[47] Kovacs M. CDI 2 – children’s depression inventory 2nd edition. Toronto: MHS (2011).

[48] Bruni O Ottaviano S Guidetti V Romoli M Innocenzi M Cortesi F. The Sleep Disturbance Scale for Children (SDSC) Construct ion and validation of an instrument to evaluate sleep disturbances in childhood and adolescence. J Sleep Res. (1996) 5:251−61. doi: 10.1111/j.1365-2869.1996.00251.x 9065877

[49] Dunn W. Sensory Profile 2. User’s manual. Bloomington: Pearson (2014).

[50] Cuzzolaro M Vetrone G Marano G Garfinkel PE. The Body Uneasiness Test (BUT): Development and validation of a new body image assessment scale. Eat Weight Disord - Stud Anorex Bulim Obes. (2006) 11:1−13. doi: 10.1007/BF03327738 16801740

[51] Garner DM. EDI-3 Eating Disorders Inventory-3: Professional manual. Odessa, FL: Psychological Assessment Resources (2004).

[52] Kaufman J. K-SADS-PL DMS-5. Yale: Yale University (2016).

[53] Lundh A Kowalski J Sundberg CJ Gumpert C Landén M. Children’s Global Assessment Scale (CGAS) in a naturalistic clinical setting: Inter-rater reliability and comparison with expert ratings. Psychiatry Res. (2010) 177:206−10. doi: 10.1016/j.psychres.2010.02.006 20334931

[54] American Psychiatric Association D. S. M. T. F D. S. American Psychiatric Association. Diagnostic and statistical manual of mental disorders: DSM-5 Vol. 5. . Washington, DC: American psychiatric association (2013).

[55] Leedham A Thompson AR Smith R Freeth M. [amp]]laquo; I was exhausted trying to figure it out »: The experiences of females receiving an autism diagnosis in middle to late adulthood. Autism Int J Res Pract. (2020) 24:135−46. doi: 10.1177/1362361319853442 31144507

[56] Leader G Hogan A Chen JL Maher L Naughton K O’Rourke N. Age of autism spectrum disorder diagnosis and comorbidity in children and adolescents with autism spectrum disorder. Dev Neurorehabilitation. (2022) 25:29−37. doi: 10.1080/17518423.2021.1917717 33934683

[57] Risk of psychiatric comorbidity with autism spectrum disorder and its association with diagnosis timing using a nationally representative cohort. Res Autism Spectr Dis. (2023) 104:102134. doi: 10.1016/j.rasd.2023.102134. Available online at: https://www.sciencedirect.com/science/article/pii/S175094672300034X.

[58] Ochoa-Lubinoff C Makol BA Dillon EF. Autism in women. Neurol Clin. (2023) 41:381−97. doi: 10.1016/j.ncl.2022.10.006 37030965

[59] KhaChadourian V Mahjani B Sandin S Kolevzon A Buxbaum JD Reichenberg A. Comorbidities in autism spectrum disorder and their etiologies. Transl Psychiatry. (2023) 13:71. doi: 10.1038/s41398-023-02374-w 36841830 PMC9958310

[60] Hosozawa M Sacker A Cable N. Timing of diagnosis, depression and self-harm in adolescents with autism spectrum disorder. Autism. (2021) 25:70−8. doi: 10.1177/1362361320945540 32772703 PMC8162135

[61] Zuckerman KE Lindly OJ Reyes NM Chavez AE Macias K Smith KN. Disparities in diagnosis and treatment of autism in latino and non-latino white families. Pediatrics. (2017) 139:e20163010. doi: 10.1542/peds.2016-3010 28557734 PMC5404727

[62] Napolitano A Schiavi S La Rosa P Rossi-Espagnet MC Petrillo S Bottino F. Sex differences in autism spectrum disorder: diagnostic, neurobiological, and behavioral features. Front Psychiatry. (2022) 13:889636. doi: 10.3389/fpsyt.2022.889636 35633791 PMC9136002

[63] Bargiela S Steward R Mandy W. The experiences of late-diagnosed women with autism spectrum conditions: an investigation of the female autism phenotype. J Autism Dev Disord. (2016) 46:3281−94. doi: 10.1007/s10803-016-2872-8 27457364 PMC5040731

[64] Di Vara S Guerrera S Menghini D Scibelli F Lupi E Valeri G. Characterizing individual differences in children and adolescents with autism spectrum disorder: a descriptive study. Front Psychol. (2024) 15:1323787. doi: 10.3389/fpsyg.2024.1323787 38476386 PMC10927760

[65] D’Mello AM Frosch IR Li CE Cardinaux AL Gabrieli JDE. Exclusion of females in autism research: Empirical evidence for a « leaky » recruitment-to-research pipeline. Autism Res Off J Int Soc Autism Res. (2022) 15:1929−40. doi: 10.1002/aur.2795 PMC9804357 36054081

[66] Fombonne E. Camouflage and autism. J Child Psychol Psychiatry. (2020) 61:735−8. doi: 10.1111/jcpp.13296 32658354

[67] Lickel A MacLean WE Blakeley-Smith A Hepburn S. Assessment of the prerequisite skills for cognitive behavioral therapy in children with and without autism spectrum disorders. J Autism Dev Disord. (2012) 42:992−1000. doi: 10.1007/s10803-011-1330-x 21818677 PMC4426203

[68] Halder S Mahato AK. Cognitive behavior therapy for children and adolescents: challenges and gaps in practice. Indian J Psychol Med. (2019) 41:279−83. doi: 10.4103/IJPSYM.IJPSYM_470_18 31142932 PMC6532387

[69] Driessen E Hollon SD. Cognitive behavioral therapy for mood disorders: efficacy, moderators and mediators. Psychiatr Clin North Am. (2010) 33:537−55. doi: 10.1016/j.psc.2010.04.005 20599132 PMC2933381

[70] Spirito A Esposito-Smythers C Wolff J Uhl K. Cognitive-behavioral therapy for adolescent depression and suicidality. Child Adolesc Psychiatr Clin N Am. (2011) 20:191−204. doi: 10.1016/j.chc.2011.01.012 21440850 PMC3073681

[71] Reinecke MA Ryan NE DuBois DL. Cognitive-behavioral therapy of depression and depressive symptoms during adolescence: a review and meta-analysis. J. Am. Acad. Child Adolesc. Psychiatr. 37:26–34. doi: 10.1097/00004583-199801000-00013 9444896

[72] Thomas JJ Wons OB Eddy KT. Cognitive-behavioral treatment of avoidant/restrictive food intake disorder. Curr Opin Psychiatry. (2018) 31:425−30. doi: 10.1097/YCO.0000000000000454 30102641 PMC6235623

[73] Murphy R Straebler S Cooper Z Fairburn CG. Cognitive behavioral therapy for eating disorders. Psychiatr Clin North Am. (2010) 33:611−27. doi: 10.1016/j.psc.2010.04.004 20599136 PMC2928448

[74] Agras WS. Cognitive behavior therapy for the eating disorders. Psychiatr Clin North Am. (2019) 42:169−79. doi: 10.1016/j.psc.2019.01.001 31046920

[75] Pantazakos T Vanaken GJ. Addressing the autism mental health crisis: the potential of phenomenology in neurodiversity-affirming clinical practices. Front Psychol. (2023) 14:1225152. doi: 10.3389/fpsyg.2023.1225152 37731874 PMC10507173

[76] Wilkenfeld DA McCarthy AM. Ethical concerns with applied behavior analysis for autism spectrum « Disorder ». Kennedy Inst Ethics J. (2020) 30:31−69. doi: 10.1353/ken.2020.0000 32336692

[77] Fieiras C Chen MH Escobar Liquitay CM Meza N Rojas V Franco JVA. Risperidone and aripiprazole for autism spectrum disorder in children: an overview of systematic reviews. BMJ Evid-Based Med. (2023) 28:7−14. doi: 10.1136/bmjebm-2021-111804 35101925

[78] Hirota T King BH. Autism spectrum disorder: A review. JAMA. (2023) 329:157. doi: 10.1001/jama.2022.23661 36625807

[79] Blankenship K Erickson CA Stigler KA Posey DJ McDougle CJ. Aripiprazole for irritability associated with autistic disorder in children and adolescents aged 6-17 years. Pediatr Health. (2010) 4:375−81. doi: 10.2217/phe.10.45 PMC3043611 21359119

[80] Hirsch LE Pringsheim T. Aripiprazole for autism spectrum disorders (ASD). Cochrane Database Syst Rev. (2016) 2016:CD009043. doi: 10.1002/14651858.CD009043.pub3 27344135 PMC7120220

[81] Wang X Zhao J Huang S Chen S Zhou T Li Q. Cognitive behavioral therapy for autism spectrum disorders: A systematic review. Pediatrics. (2021) 147:e2020049880. doi: 10.1542/peds.2020-049880 33888566

[82] Di Luzio M Guerrera S Pontillo M Lala MR Casula L Valeri G. Autism spectrum disorder, very-early onset schizophrenia, and child disintegrative disorder: the challenge of diagnosis. A case-report study. Front Psychiatry. (2023) 14:1212687. doi: 10.3389/fpsyt.2023.1212687 37575588 PMC10416439

[83] Barlattani T D’Amelio C Cavatassi A De Luca D Di Stefano R di Berardo A. Autism spectrum disorders and psychiatric comorbidities: A narrative review. J Psychopathol. (2023) 29:3−24. doi: 10.36148/2284-0249-N281

[84] Schopler E Van Bourgondien ME Wellman GJ Love SR. The childhood autism rating scale. 2nd edn. Los Angeles: Western Psychological Services (2010).

[85] Bortoletto R Bassani L Garzitto M Lamberti M Simonati A Darra F. Risk of psychosis in autism spectrum disorder individuals exposed to psychosocial stressors: A 9-year chart review study. Autism Res Off J Int Soc Autism Res. (2023) 16:2139−49. doi: 10.1002/aur.v16.11 37929657

[86] Tubío-Fungueiriño M Cruz S Sampaio A Carracedo A Fernández-Prieto M. Social camouflaging in females with autism spectrum disorder: A systematic review. J Autism Dev Disord. (2021) 51:2190−9. doi: 10.1007/s10803-020-04695-x 32926304

[87] National Institute for Health and Care Excellence (NICE). Clinical guideline [CG170]: Autism spectrum disorder in under 19s: support and management. London: National Institute for Health and Care Excellence (NICE); . (Accessed June 14, 2021). 34283415

